# Supplementary material for: Prevalence and Evolutionary Characteristics of Bovine Coronavirus in China
Source: Vet Sci. 2024 May 21;11(6):230. doi: 10.3390/vetsci11060230 (PMC11209178; doi:10.3390/vetsci11060230)
Supplement: Supplementary file 1 [file vetsci-11-00230-s001.zip › vetsci-2976827 SM.pdf]

Article

Prevalence and Evolutionary Characteristics of Bovine Coronavirus in China

Siyuan Li, Jin Huang, Xuhang Cai, Li Mao, Lingling Xie, Fu Wang, Hua Zhou, Xuesong Yuan, Xinru Sun, Xincheng Fu, Baochao Fan, Xingang Xu, Jizong Li and Bin Li

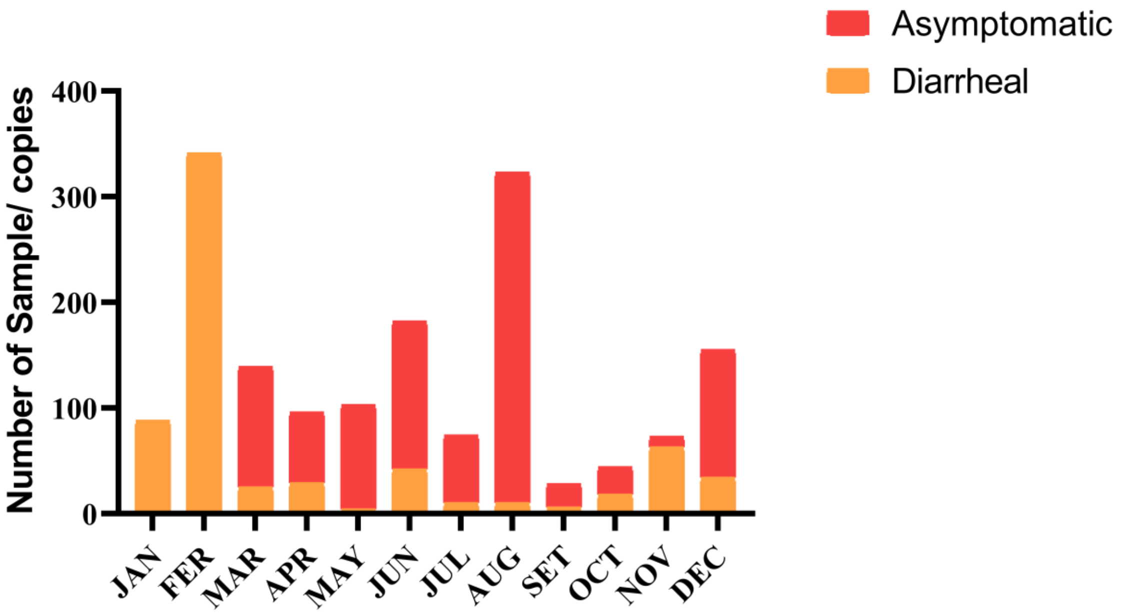

Figure S1: Monthly sample collection.
